# ‘We’re the First Port of Call’ – Perspectives of Ambulance Staff on Responding to Deaths by Suicide: A Qualitative Study

**DOI:** 10.3389/fpsyg.2020.00722

**Published:** 2020-04-21

**Authors:** Pauline A. Nelson, Lis Cordingley, Navneet Kapur, Carolyn A. Chew-Graham, Jenny Shaw, Shirley Smith, Barry McGale, Sharon McDonnell

**Affiliations:** ^1^Division of Population Health, Health Services Research & Primary Care, School of Health Sciences, The University of Manchester, Manchester, United Kingdom; ^2^Division of Musculoskeletal & Dermatological Sciences, Manchester Biomedical Research Centre, The University of Manchester, Manchester, United Kingdom; ^3^Centre for Mental Health and Safety, The University of Manchester, Manchester, United Kingdom; ^4^Greater Manchester Mental Health NHS Foundation Trust, Manchester, United Kingdom; ^5^NIHR Greater Manchester Patient Safety Translational Research Centre, The University of Manchester, Manchester, United Kingdom; ^6^School of Primary, Community and Social Care, Keele University, Keele, United Kingdom; ^7^NIHR Applied Research Collaboration, West Midlands, United Kingdom; ^8^If U Care Share Foundation, Durham, United Kingdom; ^9^Suicide Bereavement United Kingdom, Manchester, United Kingdom

**Keywords:** suicide, suicide bereavement, postvention, ambulance staff, emergency services, first responders, grief, training

## Abstract

**Introduction:**

Exposure to suicide is a known risk factor for suicide. Ambulance staff are exposed to work-related stressors including attending suicides, which may elevate their risk for mental health problems/suicide. Little is known about ambulance staff’s perspectives on how they experience these events and whether they feel equipped to respond to bereaved families at the scene of death. This study explores the perspectives of ambulance staff about responding to deaths by suicide.

**Materials and Methods:**

A convenience sample of ambulance staff recruited from one ambulance service in England. In-depth, qualitative, semi-structured face-to-face interviews conducted with nine ambulance staff (six male, three female) to explore experiences of responding to suicide. Data analyzed using thematic analysis.

**Results:**

Participants reported the experience of job-related strain including exposure to the suicide/suicidal ideation of colleagues; they described suppressing their distress despite significant emotional impact. All participants had been personally bereaved by suicide and responding to suicide was a common part of their job. They were often the first professionals at the scene, and undertook varied and often conflicting roles: negotiating with patients in crisis; informing individuals of the death of a loved one; preserving the body/potential crime scene; dealing with the intense emotional reactions of bereaved individuals. Participants reported long-term, salient memories of these events; however, there was a reported lack of acknowledgment in the workplace that suicides may be traumatic and no guidance for staff on how to cope. Opportunities to debrief were reportedly rare, and there was reluctance to access work-based liaison services. Training in how to respond to individuals bereaved by suicide was also lacking.

**Discussion:**

The study is the first to reveal the complex challenges faced by ambulance staff in responding to suicide without adequate training and support. It demonstrates the potential impact that responding to suicide can have personally and professionally on staff, and emphasizes the need for employers to support staff wellbeing in better ways. Training and postvention support could enable better coping among staff, more effective support for bereaved individuals and reduce the risk of death by suicide both in those bereaved by suicide and in ambulance staff.

## Introduction

Each year worldwide, over 800,000 people die by suicide (World Health Organisation [WHO], 2014), more than 6,000 of these in the United Kingdom (Office for National Statistics, 2019). As first responders, ambulance staff who attend deaths by suicide may be exposed to multiple such events and are expected to respond to bereaved individuals at the scene.

For over three decades there have been indications in the literature that the effects of exposure to such events on first responder staff can be distressing, leading to raised mental health issues among these professionals (James, 1988; Beaton et al., 1995; Clohessy and Ehlers, 1999; Alexander and Klein, 2001; Bennett et al., 2004). A more recent US-based survey among first responders revealed that 14% reported either moderate or severe symptoms of depression, 28% moderate or severe symptoms of anxiety, 26% significant PTSD symptoms, 31% harmful alcohol use and/or alcohol dependence, 93% significant sleep disturbance, and 34% high risk for suicide (Jones et al., 2018). Unsurprisingly, it has been known for some time that levels of sickness absence among ambulance staff are higher than among other groups of National Health Service (NHS) staff (Bennett et al., 2004) and indeed, five times the national average (NHS Information Centre for Health and Social Care, 2012). Work-related stress has been indicated as a potential reason (Mildenhall, 2012).

Exposure to suicide is a known risk factor for further suicide (Pitman et al., 2016). Research with other first responders such as the police and firefighters demonstrates that these staff may be exposed on average to 30 such events during their working lives (Cerel et al., 2018), with higher levels of suicidality than other professionals (Stanley et al., 2016). Such cumulative exposure may increase the risk of suicide among first responders (Kimbrel et al., 2016). Indeed, in recent Australian and United States studies, first responders had a significantly higher likelihood of death by suicide compared to other occupations (Milner et al., 2017; Vigil et al., 2019). In addition, suicide risk is higher in males (Office for National Statistics, 2019) and as the majority of ambulance staff are men, gender is a further group characteristic that may potentially increase their suicide risk. Research with ‘second responder’ professionals who come into contact with suicide suggests that this experience can be profoundly distressing for some (Seguin et al., 2014), that they may be unsure how to respond to the bereaved, may feel powerless to help and are unaware of other resources (Halligan and Corcoran, 2001; Foggin et al., 2016). Additionally, a lack of training in suicide bereavement has been commonly reported among professionals exposed to suicide (Foggin et al., 2016; Gibbons et al., 2019).

The perspectives of first responders about their experiences of suicide-related work are absent from the literature (Maple et al., 2019). As well as scant published literature, there is little guidance available to direct practice (National Institute For Health And Care Excellence, 2018). Consequently little is known about the experiences of front-line ambulance staff about their exposure to fatal and near fatal suicide events in the course of their work and whether they feel equipped to respond to bereaved individuals at the scene of death. The aim of this exploratory study was to illuminate the experiences of ambulance staff in relation to attendance at a suicide, including interaction with families bereaved by suicide at the place of death.

## Materials and Methods

As the aim was to explore the experiences of ambulance staff from their own perspectives, the study took a qualitative approach.

Ethical approval was obtained from an NHS research ethics committee in North West England (reference 11/NW/21047). A convenience sample of self-selecting participants from one ambulance service in England was recruited to take part in semi-structured exploratory interviews. Those ambulance staff who could recollect their experiences of responding to a suicide (involving contact with individuals bereaved by suicide at the place of death) were eligible to take part.

Information about the study was advertised in a regional staff bulletin, on the staff intranet and by poster displayed on ambulance station notice boards. Potential participants were invited to contact the research team directly by email or phone to express their interest in taking part. Further information about the study was provided to those expressing interest in a more detailed information sheet, verbal clarification was also offered and staff had the opportunity to ask questions before deciding whether or not to participate.

If participants confirmed their willingness to take part in the study a convenient date, time and interview location was arranged. Participants provided informed, written consent before any data collection took place in in-depth, semi-structured, face-to-face interviews in their homes or workplaces. A topic guide developed from the literature guided the interviews to explore experiences of responding to suicide (see Table 1).

**TABLE 1:** Interview topic guide.

**Questions**	**Prompts**
Tell me about your experiences of attending a suicide/suicide attempt as part of your job.	Rare/common occurrence?
How does attending a suicide impact upon you?	
Describe your experiences of responding to bereaved individuals at the scene of a death by suicide?	Challenges/opportunities?
How equipped do you feel to respond to bereaved individuals in these circumstances?	Helpful/unhelpful aspects?
What workplace support is in place to help you with suicide-related work and what else could help?	Formal/informal support?

Interviews were conducted with participants in February 2014. Interviews were audio-recorded, transcribed, anonymized and exported to NVivo 12 software for data management and coding (QSR International Pty Ltd., 2018). Data were jointly analyzed by the first and second authors using a thematic analysis approach to enable production of pre-determined and new ideas (Braun and Clarke, 2006), focusing on similarities and differences in participants’ accounts. Data were jointly discussed and refined before key superordinate themes were identified.

## Results

### Participants

In total, nine ambulance staff (six male, three female, all white British) took part in in-depth interviews lasting between 50 and 110 min. All participants were experienced staff, working in the ambulance service between 8 and 28 years (see Table 2 for sample characteristics).

**TABLE 2:** Final sample characteristics.

**Participant**	**Gender**	**Professional role**
P1	Male	Paramedic
P2	Female	Paramedic
P3	Female	Advanced technician
P4	Male	Paramedic
P5	Male	Paramedic
P6	Male	Advanced paramedic and station manager
P7	Female	Paramedic
P8	Male	Paramedic
P9	Male	Paramedic

#### Key Themes

Three key theme sare presented using illustrative data extracts that capture participants’ perspectives on responding to suicide: (1) a profession under strain; (2) responding to suicide in a professional capacity and; (3) lack of workplace support following exposure to suicide. Participants’ names and some of the detail of extracts are anonymized to protect the identity of those taking part.

##### A profession under strain

###### Lack of understanding of ambulance staff’s role and low professional kudos

Ambulance staff described themselves as a profession under strain. One source of pressure was a perceived lack of understanding about the role of ambulance workers (and in particular of the challenges they face in carrying out their duties), among both the public and other health professions. This was compounded by the simultaneously high expectations of service users in relation to what they imagined ambulance staff could offer at the scene of traumatic incidents such as suicides or suicide attempts:

…*healthcare professionals don’t know what we do; GPs [primary care doctors] haven’t a bloody clue what we do.* (P5, male)…*I have to say, in situations like [suicide] or any other situation, people’s expectations of the ambulance service is phenomenal.* (P3, female)

Many felt that even friends and neighbors did not understand their job and misunderstood them as emotionally detached when responding to intensely traumatic incidents:

*They just seem to think we’re on autopilot and*… *nothing bothers you, nothing affects you*… *I think they see us as real hard*…*very hard people and we’re not*… (P7, female)

In relation to the many other NHS professions, ambulance workers reported feeling demoralized, forgotten and neither valued nor respected by colleagues from other disciplines:

*We are the forgotten*… *the forgotten group the ambulance personnel.* (P9, male)

*We’re the service what everybody uses and abuses.* (P7, female)

Participants viewed themselves as somewhat unpopular with other parts of the NHS such as accident and emergency (A&E) and psychiatric departments, as a result of being seen to be the generators of work for other staff:

*You walk in with a patient and [A&E staff] look at you and – ‘you’re here again with another one?’ You know, it’s like ‘well, what do you want me to do, go round the block?’* (P2, female)

*I won’t say they don’t like us, but basically, we’re the person who brings them the work. So, if they’ve got a department that’s full or nearly full and two or three ambulances pitch up outside A&E, obviously they’re going to be thinking ‘well is there somewhere else they could’ve gone?’* (P5, male)

Participants stressed that these difficulties could intensify with A&E or public services outside the NHS such as the police, if for example a suicidal patient had mental health issues or had consumed alcohol. Such responses served to increase ambulance workers’ sense of isolation because the options for channeling patients appropriately were significantly narrowed in these cases; some described the added strain of often having to take up defensive positions with other services to handle these challenges:

*In regard to the psychiatric side of things you are limited at what you can do because*… *once they’ve had alcohol nobody seems to want to touch them*… *we haven’t got an avenue to go down, about half the time*. (P5, male)

*You learned very, very quickly, to stick up for yourself, whether it was clinically, personally, professionally, you learned just to stick up for yourself*… *and I think*… *that’s the case, because paramedics are the lowest common denominator.* (P8, male)

The high expectations of the ambulance service reported by participants combined with a perceived lack of understanding of their role and little respect from colleagues inside and outside the NHS, contributed to experiences of job-related strain among ambulance staff.

###### Professional and personal bereavement by suicide

A significant additional source of strain for staff was losing colleagues to suicide during their career in the ambulance service. Indeed all participants reported having lost a colleague to suicide at least once and often multiple times. Knowing colleagues who had either attempted or died by suicide was therefore not uncommon:

*So I think roughly in that space of time [10 years], three of probably the first five paramedics in the country*… *are all dead or tried to take their lives.* (P6, male)

One participant described going on shift, to be informed that a member of his team had died by suicide. He and his colleagues felt they had no option but to conceal their distress and continue working, regardless of their closeness to the colleague who had died:

…*we’d come in on the shift and we were told, and basically you manned your ambulance and went out. If you didn’t go*… *then there’d be no ambulances going out there. So you’re just*… *well, like robotic. You just went out.* (P5, male)

Participants often lived in the geographical area in which they also worked. As a direct result there would be occasions when, in responding to an incident, the person who had died by suicide was either a colleague or a family member of a colleague; such incidents were extremely distressing for crew members:

*Not sure what triggered it off, but he*… *took a massive overdose and died, and we resuscitated him.* (P6, male)

*Our crews went out to [colleague’s suicide] and that, as a group, affected us really badly.* (P8, male)

Some staff had, in the course of their work, been charged with informing colleagues that members of their own family had attempted or died by suicide; others remarked that suicidality among the profession was fairly commonplace:

*[Colleague 1] was at a job, and [Colleague 2] went. [He] was the officer running [the region] at the time and*… *it fell to him to go and get [Colleague 1] and tell him that [family member had made a suicide attempt], and then say to him*… *you need to step aside, the crew is taking over*… (P3, female)…*when you’re talking to different members of staff there’s been a couple that have talked to me, and they’ve sort of been at a very low ebb*… *you know*… *I could become a midlife statistic myself.* (P1, male)

In addition to losing colleagues to suicide, some participants had family members who had attempted or died by suicide; one had lost a close family member and attributed his own attempted suicide to this loss. Despite being bereaved by suicide, with suicidal ideation, on returning to professional duties he was expected to attend suicides as a first responder.

Exposure to the suicide or suicidality of colleagues was an added strain for ambulance workers, yet staff habitually learned to suppress their feelings of distress and continue their work despite significant emotional impact.

##### Responding to suicide in a professional capacity

###### The impact on ambulance staff of attending suicides

Ambulance staff were routinely called to suicides or suicide attempts as a *‘daily’* (P7, female) part of the job, involving a range of circumstances. Staff were often the first professionals at the scene:

*It’s what we get called to, to be honest, you know, suicide goes on, we’re going out to it, so if somebody’s dying, finding them*… *whether it would be by pills, other means or anything*… *we’re the first port of call. I’ve done at least four in the last 10 months probably myself*… *but we get that many.* (P1, male)

*Well who deals with it? Well, it’s a sudden death, we do, and the police – long before you get to GPs, mental health, social services*… *it’s us and the police, no one else touches [suicide].* (P8, male)

Responding to a suicide or a suicide attempt could strongly impact ambulance staff and be emotionally demanding, more so than attending the scene of accidents or other traumas:

… *there’s quite a few [suicides] where it’s been really bad. the worst one I had was the [homicide-suicide] over in Locality 2 where [parent killed family and self]. That was one of the worst ones*… (P5, male)

*I can put [suicide] quite high up ’cos*… *I find that harder to deal with than trauma. Trauma to me is just an everyday thing what we go out to. I can deal with that – that’s my job, that’s what we do*… *seeing somebody hanging*… *it’s the most horrendous thing ever to see somebody hanging. I mean for me that’s the worst job ever*. (P7, female)

Attending a suicide could evoke personal feelings of distress and vulnerability for crew members, particularly where staff identified in some way with the deceased (for example if the person who had died by a similar age to one of their children or other relatives):

*How*… *could you imagine your own child dying, you know how must that feel? It’s unthinkable isn’t it, and I think for some staff we have children that are at home that are probably the same age as somebody that you’ve just dealt with.* (P4, male)

*People*… *will come back to the station*… *they ring home and make sure everyone is all right, you know, because that’s how it affects you.* (P2, female)

Participants reported that it was difficult to forget what they saw in the course of their work. Some described long-term, salient memories of exposure to suicides that they could recall in fine detail, often years after the event; the most traumatic events were often etched in their memories:

*Suicide[s] they always stick*… *there’s always a thing which stays in the back of your mind. I could take you to the first suicide [I attended]. It’s not far from here, I could take you to where we found him in the car. I can describe how we got him out of the car, how he was positioned, his size and everything.* (P9, male)

*It would be*… *probably about 1990, something like that, ’91. It was a nice summer’s day like this*… *all the detail yeah*… *you do get flashbacks.* (P5, male)

Suicides could *‘haunt’* (P9, male) thoughts and interfere with sleep and normal activities:

…*it was horrific; it was like a horror movie! [The deceased] basically, the body had exploded*… *the belly and everything*… *I couldn’t sleep. I just constantly*… *it was like a horror movie, I just kept seeing this [person]*… (P7, female)

*I wouldn’t believe anybody that had been to, whether it was an adult or a child suicide*… *if they said that it didn’t affect them because it always affects you*… *I’ll go to bed and I’ll dream about it and I’ll have strange dreams and I’ll think about it*… *[suicide] affects you mentally in some other way whether you think that you’re over it or not.* (P4, male)

Participants described exposure to multiple suicides in the course of their work, incidents which could be personally distressing. Attending suicides could evoke salient and detailed recollections that could impact on normal functioning and endure over the long-term. Exposure to suicide was said to induce subconscious trauma of which staff could be unaware.

###### Conflicting roles at the scene of suicides or suicide attempts

Ambulance staff undertook varied and often conflicting roles when responding to fatal and non-fatal suicide attempts. One role was to deal with individuals who were experiencing suicidal thoughts or who were actively suicidal. Professionals reported feeling in danger in such situations and often ill-equipped to respond appropriately to individuals in mental crisis:

*I remember going to a woman who*… *said she wanted to kill herself, and I remember*… *talking with her and she was very distressed*… *and I remember looking round and taking things like pencils away*… *but*… *she ran into the kitchen and*… *she pulled out this massive*… *serrated bread knife and because she went like that [brandishes knife]*… *stupidly, I put my hand round the serrated knife like that and I just.* (P3, female)

*This is what [patient] was telling me last night – ‘I’ve had to come out of the flat because I’m searching for a rope and I know that I’m going to kill myself.’ Well, I just sat there and thought ‘God, what am I gonna do here? What*…*?’ Didn’t want to die, but he said he’d got somebody sitting here telling him to go and do it*… (P7, female)

Ambulance staff described fulfilling several different professional roles at once in the course of responding to suicide attempts. For example they could be functioning like ‘*social services’* (P3, female) by taking on a welfare role with some patients and assuming duties that they perceived to more appropriately sit with other public services:

*I’ve googled things for people – where the nearest Citizens Advice is or whatever*… *‘cause sometimes they’ve got money issues as well and the landlord has wanted them out*… *and years ago the police used to take the violent ones, but now*… *because [police are] having death in custody*… *they’re washing their hands of it*… *and*… *we mop up everything, from a drunk in the street who’s just been thrown out of a pub or whatever [to] the people who can’t get GPs*… (P5, male)…*we got a girl who was*… *trying to do herself in, commit suicide, and we had to pull over because*… *she was trying to*… *get out the back door as the motor’s moving. So, there are two of us in the back trying to hold her down*… *and we were trying desperately to stop her*… *and all she wanted to do was jump out the doors and run in front of a car. But sometimes the police*… *they sometimes try and get out of that by saying ‘we’ll follow in the car right?’* (P1, male)

Ambulance staff as first responders had to multi-task at the scene of a suicide or suicide attempt, performing resuscitation, dealing with relatives and transporting patients to hospital. While non-fatal suicide attempts could be seen by some as less emotionally draining because the professional could actively focus on saving a life and treating injuries, such events often brought other challenges and potentially multiple patients at the scene to deal with:

*I’ve been to a child death before where a child has been hanging and you know, you’re trying to deal with the parent but you’re trying to resuscitate the child, and for those first 20, 30 min*… *I’m sorry, [parent] can have some involvement but my main*… *your duty of care is to that child.* (P3, female)

*It’s challenging, but because you’ve got a patient to treat, [non-fatal suicide attempt is] not as upsetting – daft as it seems – because you’re focused on the injuries and you can*… *you’re in automatic pilot because*… *don’t forget, you’ve got other people there who are potentially patients, i.e., the driver of the train ‘cause he’s just seen somebody jump in front of him so he’s emotionally upset. And it can bring on heart attacks, it can you know, the stress.* (P5, male)

In relation to a potential suicide, ambulance staff described being expected to fulfill two conflicting roles. Initially arriving with the aim of saving life, when a person was dying by suicide (or was already dead), staff described immediately becoming custodians of a potential crime scene. It was reported that when a person had died, these scenarios may be a low priority for the police, meaning long waits at the scene of death before police attend:

…*the police have got to be there, the body has got to be taken down after forensics have been, and then you can deal with the family after*… *so it then potentially becomes a crime scene, because we don’t know if it’s a suicide or whether it’s an assisted suicide*… *so we have to inform the police*… *it’s not a priority [for the police], because the person is deceased, so we can wait until the police turn up and then nothing can be done.* (P2, female)

*Because we’re now being asked to do the police’s job, and*… *the police are taking 40 min to get to a job*… *it’s a potential crime scene.* (P8, male)

These were potentially volatile situations in which staff could be exposed to the distress of bereaved families over a prolonged period. Staff perceived that denying people access to the body of their loved one (when they wanted to care for the deceased) could be experienced as an insensitive and even inhumane act, risking violence or verbal abuse from families. Thus, preserving the potential crime scene without adequate support, often induced feelings of helplessness in ambulance staff. As well as shielding the body of the deceased from families, conversely, staff felt it was their duty to shield families from the body. Far from being insensitive to the feelings of bereaved families, this was in an effort to protect the emotional wellbeing of the bereaved by preventing visual exposure to the deceased:

*[Deceased by suicide had] taken a massive overdose*… *but, he’d been there a week and he was*… *hanging down the settee and we couldn’t recognize the features*… *but*… *then the family had been told, and the family came down, and we couldn’t let them in and see him as he was, because they wouldn’t have recognized him*… *so we were trying to keep the family*…*they wanted to come in*… *luckily the police turned up, else we wouldn’t have been able to stop them.* (P2, female)

*The police come along and they become so clinical*… *[husband] knows his wife is still hanging in the garage, all he wants is [for her] to be able to be laid at rest. And, I’m there for another, what, hour and a half, and she’s still there, because [police] have got to wait for the forensics to come, and take pictures and all this. Then, they call for the fire brigade to come and get her down, so then you’ve got six firemen coming in, [husband has] seen his wife in this state, they’re traipsing in and out. his son comes, his son wants to see his mother but he can’t*… (P9, male)

One ambulance worker who had been bereaved by suicide himself felt conflicted about what he described as the *‘litigation’* culture that dictated the leaving of bodies in place after a death by suicide. He felt there should be more respect for bodies of the deceased and for the bereaved and described his own attempts at practically demonstrating such respect at the scene of suicides:

…*it’s having the respect for the dead as much as the living*… *like when I go out to a [suicide] if there’s no reason for the [deceased] to be where they are, I want to put them so that they’re at rest. So, if I can get them into a bed, or I can get them onto the settee and*… *put a blanket on them so they look at peace, or the*… *relative who wants to cuddle with them, and they want to lie with them, I haven’t got a problem with it.* (P9, male)

Ambulance staff described conflicting roles and duties at the scene of suicide or suicide attempts; some that they felt ill-equipped to handle and others that they believed could be more appropriately managed by other public services.

###### Dealing with the reactions of people at the scene

As ambulance staff were often the first professionals at the scene of a suicide, they were tasked with informing the family or friends of the deceased that a loved one had died. This meant that staff had to contend with often *‘volatile’* (P1, male) situations and deal with the intense emotions/reactions of bereaved individuals, sometimes more than one. Staff could encounter extreme distress, anger and even violence among the bereaved, mixed with strong feelings of guilt or shame that those close to the deceased had not been able to anticipate the death:

…*by the time the [family] arrived on scene, they saw [deceased by drowning*… *and that was a very difficult job. you’ve got [family] and they turn up at the scene*… *and they were in pieces.* (P8, male)…*[wife of deceased] just completely blew her lid*…. *it was an emotional not an aggressive state, but you know, she was trying to move the body*… *but then once the daughter had calmed down, she did then warn us that the son was on the way and he would be aggressive. She just said he would flip, so we had to leave the property*… *because she said*… *he’ll start throwing and smashing things around the house and he won’t care who’s here.* (P2, female)

When a person had already died, staff could feel ‘*absolutely helpless’* (P7, female), and often felt blamed by bereaved individuals for not being able to bring the person back. This was compounded by having to complete paperwork after a death, involving questioning the deceased relatives and friends that some staff felt was inappropriate:

…*one of the things that the family want you to do*…*‘why aren’t you doing something?!’ The fingers pointed – ‘why aren’t you trying to get my loved one back?’, even though they probably realize that their loved one’s gone. We’re not just standing with our hands in our pockets, but we’re trying to calm the situation*… *we can’t do anything, because there’s nothing we can do. But they vent frustrations to us because it looks like we’re just standing there doing nothing for their loved ones, and our main role is to support life, and keep people alive, and the family can’t understand that.* (P1, male)…*we’ve got to go back to the*… *relatives and the parents of [person who has died by suicide] and say, can I start asking you a few questions now please? [The] police [should carry out this function], I personally think we’ve been through enough with the resuscitation and*… *we have to enter the property again and come back into the situation, then start again*… *it’s a different role then*… *because the person is really now deceased and then you’ve got to start off bombarding the relatives with questions*… (P2, female)

In addition to handling the reactions of the deceased’s family and friends, ambulance staff were also called upon to deal with members of the public and other colleagues who may need support at the scene of a suicide. Staff could feel overwhelmed and often helpless when faced with these multiple responsibilities:

*I went out to a [violent death by suicide of a young person]*… *I’d got a policeman saying he was ready to vomit, I’d got a student paramedic with me who couldn’t cope with it, and I’m trying*… *to deal with [colleagues] and deal with*… *the [person] was dead, but [unconnected members of the public were caught up in the incident]*… *I couldn’t, I didn’t know what to do*… (P7, female)…*we had*… *a young girl, she was a trainee [ambulance] technician. She [was] just out on her 1st day as a tech, and that was her first sight of a dead body, and she couldn’t believe how the family reacted, and she couldn’t cope, she broke down crying. We had to deal with her because*… *the hysterics of what was happening, the daughter and then the mother coming in*… (P2, female)

At the scene of a suicide, ambulance staff reported being regularly tasked with dealing with the distress of bereaved families and members of the public/colleagues who were also present. Circumstances could be volatile when a loved one was unable to be revived, with the bereaved often venting their frustration on staff who felt powerless to help.

##### Lack of workplace support following exposure to suicide

###### Lack of formal acknowledgment of the potential impact of suicide on staff

Despite the demands placed upon ambulance staff when attending suicides there was a reported lack of acknowledgment in the workplace that this work may be traumatic and no guidance for staff on how to cope. While the opportunity for taking a break or de-briefing after traumatic incidents including suicides was reportedly offered in theory, in practice it was said to rarely happen due to time constraints, staff shortages and *‘by the clock’* (P5, male) working:

*In principle, they have it, if you want to go home you can, but I think in reality very few people ask for it*… *and I don’t think [service managers] want you to do it, because they want you to stay available*… *it wouldn’t be encouraged*… *they’ll say ‘can we clear now for the next [Category] A?’ they want you clear for that. So in terms of me asking for counseling and time out, they’ll be watching that.* (P8, male)…*from the moment I put on the radio ‘book clear’ [after a suicide] – that’s when you become available – and it literally is a bump on the screen, you’ve got another job to go to*… *there’s no ‘oh are you okay?’*… *because*… *we’re just stretched to the limits, there’s not enough of us, there’s not enough vehicles out on the road, and you’re just literally being bombarded.* (P9, male)

In addition to time-pressured working which could discourage staff to ask for support, there was also a reported culture of *‘manning up’* (P5, male) which was perceived to signal in more imperceptible ways to staff (even those personally bereaved by suicide), that needing support was not be encouraged:

…*you can ask for time out after a bad job*… *and I would suspect very few ask for it. And even if you did ask for it, [management] would probably think you were pulling a fast one*… *I suspect.* (P8, male)… *the unfortunate part [is]*… *if you can’t cope with the stress, you shouldn’t be in the job. There’s no such thing as stress in the ambulance [service].* (P9, male)

There was said to be a *‘stigma’* (P2, female) around asking for help and a ‘macho’ culture which encouraged the use of ‘*black humor’* (P4, male) and, even for female members of staff, *‘blokey things’* (P3, female) as coping mechanisms in situations of high stress. The prevailing ethos was to suppress internal distress, ‘*shut it down’* (P8, male), and force oneself to continue working regardless of feeling traumatized:

…*there’s a lot of new staff coming on*…. *and some of them might be a little bit nervous to say ‘I’ve just seen something and I can’t deal with that’*… *and then are they thinking ‘well I’ll try and work through*… *and think I can deal with this’.* (P1, male)*I wouldn’t ring the SALS [staff advice and liaison service] up to get any help because I feel that people would say she knows what the job’s all about*… (P7, female)

There were some isolated reports of workplace support being offered to staff who were distressed by attending suicides or being bereaved by suicide, however, the overriding representation was one of the absence of easily accessible support after trauma, including the suicide of colleagues. There was no official workplace guidance or protocol on how to respond to suicides or how to care for staff who may be affected by suicide; the onus was on staff themselves to look out for signs of distress in other colleagues:

…*there’s nothing we’ve been given that says, if you go to this job and then you come back in [you] have somebody to speak to.* (P2, female)…*we’d have to sort of rely on SALS [staff advice and liaison service] members and other staff members to keep an eye out on people who’ve gone to things which may trigger them, such as suicide.* (P3, female)

Even in the case of informing co-workers that a loved one had attempted suicide or taken their own life, there was no protocol in place to guide this eventuality. In light of this staff talked about coping informally among themselves, though relying on the support of colleagues alone could be problematic. The availability of peer support was affected by time pressures and increasing demand on the service which saw staff working out on the road in isolation for long periods rather than being able to connect with co-workers at ambulance stations; staff felt unsupported even when they were tasked with responding to major critical incidents such as a homicide/suicide:

*Years ago you used to do a job and*… *your crew members would be coming back to station, control would ring the station if there was a crew there – ‘look the crew are coming back they’ve had a bit of a nasty job, either a fatal, or a child, or a suicide*… *[just] giving you the heads up’. So as soon as they come – cup of tea, and you would sit down and you’d have a chat, and you used to self-debrief*… *and now*… *you don’t even see stations because we haven’t got stations no more.* (P9, male)

*[Deceased]*… *had*… *[killed family members and self]. There was nothing in place [for staff] except just looking after yourselves and looking after your crew mates*… *that’s your support, is your mate. but if you haven’t got a good mate*… (P5, male)

The potential impact on staff of attending multiple suicides was reportedly not acknowledged and there were barriers that discouraged staff from help-seeking when needed. In the absence of formal structures and guidance on how to support staff in relation to exposure to suicide, workers relied on informal peer support, though this could be problematic.

###### Reluctance to access work-based support

There was a reported reluctance among staff to access work-based liaison services that were described by some participants as merely *‘lip service’* (P8, male) or *‘tick box’* (P3, female) because they were purportedly not used by staff:

…*[what’s] available*… *is not always accessed*. (P6, male)…*[staff] don’t use it. I don’t know many people that do use it.* (P7, female)

Reasons for this reluctance included worries about confidentiality and a lack of expertise among those who staffed the service. The staff advice and liaison service (SALS) was described as being staffed by ‘*a bunch of volunteers’* (P3, female) from within the same service as potential users, eliciting concerns about possible disclosure of private information among co-workers. Participants suggested that they would be more likely to use a service that was more independent:

…*you think well, you know, if I talk to him it will go round the station, it’s not necessarily confidential, so it would be better if you knew there was*… *somebody confidential or a confidential service that you could speak to.* (P4, male)…*the people who do the SALS, I know it’s all confidential, but*… *you know them and they work on the road*… *and*… *that’s the reason why I don’t tend to use it*… *I’ve got this fear of going to one of them and it not being strictly confidential. If I didn’t know them and it was a separate organization, I’d quite happily go and see them.* (P7, female)

There was also concern that *‘[the] ambulance service doesn’t really know how to cope with mental health*’ (P3, female) with an accompanying lack of confidence in the expertise among SALS volunteers. The service was perceived as unable to handle staff who were suicidal:

*I’m worried about the training, because all [SALS] are is ordinary ambulance people*… *who’ve done a bit of a course. And*… *they care, and they make the effort and obviously they’ve done the training, but I don’t think they’ve done proper training.* (P5, male)…*if it did come to the crunch that I did decide to take my life, I’ll be honest with you I wouldn’t ring [SALS], because I don’t*… *I really don’t think that they would be able to cope at that point.* (P9, male)

Services for ambulance staff who may need support after exposure to suicide were seen as somewhat tokenistic. There was a reported reluctance among staff to access such services because they were typically staffed by volunteers from within their own ranks who were perceived to lack expertise in mental health and suicidality; there were also concerns about confidentiality and a preference for the provision of independent staff support services.

###### Lack of training in responding to suicide

Participants reported that they had *‘no training*… *whatsoever’* (P7, female) in how to respond to relatives at the scene of suicides and described being in the position of *‘feeling your way’* (P5, male) in these situations. There were reports from staff of feeling helpless in these instances leading in some cases to a lack of engagement with the bereaved:

*It’s a horrible feeling because you don’t know what to say to them. What can I say to them that’s gonna make them feel any better*… *what the hell do I say to these people here? I don’t know what to say.* (P7, female)…*you can’t say anything, you can’t do anything*… *my strategy is say as little as possible*… *there’s nothing you can say*… *‘you’ll be all right, or, it’s okay, it’ll pass’ because it won’t pass. And it won’t be all right, [they’ll] mourn them for the rest of their life. So I say as little as possible*. (P8, male)

Some staff commented that the lack of training in how to respond to people who were bereaved by suicide was surprising because attending these kinds of deaths was such a common part of the job. Additionally, staff had no knowledge of how to respond to colleagues who had themselves been personally bereaved by suicide:

*[Colleague]*… *didn’t realize how my [close family member] had died at first until I broke down and told her. And then she said like*… *she didn’t know how to cope with it*. (P9, male)…*it was all very much, you know, supporting each other in the duty room because we’d lost one of our colleagues, he’d killed himself.* (P6, male)

It was felt that the ‘*focus should be on supporting ambulance staff’* (P8, male) in how to respond to the bereaved by suicide appropriately. It was reported that *‘accredited’* (P5, male) training in this area that was recognized by a paramedical professional body would be beneficial for ambulance staff to address gaps in knowledge and skills and confidence:

*I dealt with [suicide]*… *the only way I knew how to. But, sometimes you, you get blinkered*… *[and] if somebody else says ‘oh well, have you thought of it that way? Oh, never thought of that.’ – so*… *[training] might*… *you know, broaden the horizons to say, yes this is another way of doing it. Because if I go to a suicide*… *I can only deal with it how I personally dealt with it*… *because I don’t know how else to deal with it.* (P1, male)

*I think any training will give staff confidence and, you know, you’re never going to bring that person*… *back. but relatives and those that have been bereaved want to talk about it*… *you know, they want to talk about*… *what you’ve done, what you found, you couldn’t do anything because of this, and you know, you did this, because it’s all part of that grieving, I suppose isn’t it?* (P4, male)

Participants reported a lack of training in how to respond to people bereaved by suicide (including colleagues) and feelings of helplessness which led some to disengage completely from families at the scene of death. Training that was endorsed/accredited by a professional body was viewed as potentially beneficial in equipping ambulance staff with the skills and confidence to respond more appropriately to the bereaved at the scene of fatal suicides.

## Discussion

### Key Findings

The reportedly high expectations of the ambulance service among the public combined with a lack of understanding about their role, and a perceived lack of respect from colleagues inside and outside the NHS contributed to participants’ experiences of job-related strain. Exposure to suicide or the suicidal ideation of colleagues was an additional intense source of strain for ambulance workers; staff reported learning to suppress their distress to continue working in their high-pressure professional role despite the significant emotional impact they experienced.

Participants described exposure to numerous suicides in the course of their work, incidents which they found extremely distressing. Attending suicides could evoke salient and detailed recollections of violent and distressing deaths by suicide that impacted on normal functioning and could endure over the long-term. Some participants reported feeling traumatized when they responded to such incidents, but felt under pressure to suppress their emotions, due to the lack of support within the workplace; however, exposure to suicide was also said to induce subconscious levels of trauma that staff may not be immediately aware of and that may result in delayed stress reactions/mental health issues. Ambulance staff described conflicting roles and duties at the scene of suicide or suicide attempts which could be difficult to manage. Staff reported feeling ill-equipped to carry out some of these duties, such as managing people behaving dangerously or in mental health crisis; some staff believed that these situations could and should be managed by colleagues with more appropriate expertise such as the police or mental health/social services professionals. At the scene of a suicide, ambulance staff were tasked with dealing with the distress of bereaved families and members of the public/colleagues who were also present. Circumstances could be volatile, particularly when resuscitation was unsuccessful or when they denied families access to the body. Staff were fearful that families bereaved by suicide would vent their frustration on individual ambulance workers.

The impact on staff of attending multiple suicides was reportedly not acknowledged in the workplace and there were barriers that discouraged staff from help-seeking when needed. In the absence of formal structures and guidance on how to support staff in relation to exposure to suicide, workers relied on informal peer support, though this could be problematic. Work-based services offered for ambulance staff who may need support after exposure to suicide were seen as somewhat tokenistic and there was a reported reluctance among staff to access such services. Reasons for such reluctance included that services were typically staffed by volunteers from within workers’ own ranks and perceptions that volunteers lacked the necessary expertise in mental health and suicidality to appropriately handle staff’s needs; there were also worries about the confidentiality of volunteer services and a preference for the provision of an independent source of staff support. There was also a reported a lack of training in how to respond to people bereaved by suicide (including colleagues) and feelings of helplessness which led some ambulance staff to disengage completely from interactions with the bereaved at the scene of such a death. Training that was endorsed/accredited by a professional body was viewed as potentially beneficial in equipping ambulance staff with the skills and confidence to respond more appropriately to the bereaved at the scene of fatal suicides.

### Contribution to Current Understanding

While qualitative approaches have previously examined paramedic experiences of providing care for people who self-harm (Rees et al., 2017), to the authors’ knowledge this study is the first to illuminate in depth the first-hand perspectives of ambulance staff in relation to attendance at a suicide, including interaction with bereaved families and individuals at the place of death. These qualitative insights therefore build on and extend existing knowledge to reveal how multiple and intense sources of strain and experiences of attending cumulative traumatic events such as suicides may contribute to the raised levels of mental health morbidity, distress, suicidality, and sickness absence among first responders seen in existing quantitative studies and survey data (Beaton et al., 1995; Clohessy and Ehlers, 1999; Alexander and Klein, 2001; Bennett et al., 2004; Mildenhall, 2012; Pitman et al., 2014; Kimbrel et al., 2016; Stanley et al., 2016; Milner et al., 2017; Jones et al., 2018; Vigil et al., 2019).

The accounts reveal the complex challenges faced by ambulance workers in responding to suicide, i.e., exposure to multiple suicides (including witnessing violent suicides and attending the scene after a homicide-suicide); negotiating with suicidal individuals without adequate training/support; dealing with distressed and sometimes violent individuals bereaved by suicide without appropriate training (including fulfilling the potentially conflicting role of health professional and guardian of a potential crime scene by denying bereaved families access to the deceased); responding to distressed colleagues without adequate training (including caring for trainees at the scene of death); being in potentially dangerous situations and fearing for their own safety; feeling professionally isolated, unsupported by employers, undervalued by other services and sometimes powerless to help the people they are responding to; working under relentless time pressure with blurred boundaries between their role and the roles of other public service professionals.

This study offers a first step in understanding the impact that responding to suicide has personally and professionally on ambulance staff. Given that work-based support services were reportedly not fit for purpose, there is a need for employers to support the wellbeing of first responder staff in better ways so that this high risk group (staff exposed to suicide) might respond to another high risk group (individuals bereaved by suicide) by delivering high quality care and support in profoundly distressing circumstances (McDonnell et al., 2020). In England, policy has recognized that better information and support should be provided to all those bereaved or affected by suicide (HM Government, 2012) and that such postvention support (including for staff) is a fundamental tenet of suicide prevention (HM Government, 2019; NHS England, 2019); likewise, postvention support is a key aspect of suicide prevention internationally (World Health Organisation [WHO], 2018; Andriessen et al., 2019a, b).

In line with its duty of care to staff, the NHS Staff and Learners’ Mental Wellbeing Commission report in England (Health Education England, 2019) emphasizes the importance of promoting and supporting the wellbeing of NHS staff and all those learning in NHS settings. The promotion of self-care in the workplace for staff is therefore a key objective in the report, with recognition that particular professional groups such as ambulance workers and other first responders are at significant greater risk of stress and trauma (including suicide). The report’s recommendations in relation to self-care for learners are particularly relevant to supporting ambulance staff trainees who, as evidenced in this study, may be attending fatal and near fatal suicides under the supervision of staff who may themselves be distressed and feeling ill-equipped to support their own and others’ wellbeing in these scenarios. The ‘macho’ and somewhat unforgiving working culture and dispassionate management style reported in this study are in direct contrast to the ethos of respect and recognition for staff espoused in the staff mental wellbeing report (Health Education England, 2019). In other countries, job-related factors such as emotional exhaustion and bullying have been indicated as potential factors in first responder suicidal ideation (Sterud et al., 2008). It is hoped that in England at least, the recent staff wellbeing report (Health Education England, 2019) will help to drive the required change in culture so that mental health problems experienced by health professionals as a result of their occupation are avoided. Improving staff and learner wellbeing may also help to improve morale, lower the number of work days lost to services, potentially reduce the suicide rate in this high risk profession.

The study suggests that when afforded the opportunity (for example, being allowed time out for reflection and/or access to colleagues who can listen and empathize), ambulance staff attempt to support each other in times of high stress. It is known that informal support and camaraderie among colleagues is a protective factor against adverse mental health and suicide risk (Stanley et al., 2016), however, the current study suggested that the organization of ambulance services can block rather than enable this. The aim of workforce remodeling to reduce issues of staff isolation and lack of contact among team members found in the staff wellbeing report is laudable and very relevant to ambulance services (Health Education England, 2019).

Protocols are needed in the workplace to guide both the response of ambulance staff to suicide (including dealing with the deceased and the bereaved with sensitivity and respect) and the response of employers to staff who may have lost colleagues to suicide and/or attend the suicide of a colleague. Given that ambulance staff may be at the scene of death for a prolonged period before other services arrive in instances of suicide, evidence-based training in critical incident management (Everly and Mitchell, 1997; Maple et al., 2019) is needed. Other qualitative research (Foggin et al., 2016) has demonstrated the extent to which even second responders such as GPs find facing the intense distress or anger of people bereaved by suicide difficult; it has also been emphasized that first responders need continuous training in suicide intervention and skills for coping with trauma-related stress (Lygnugaryte-Griksiene et al., 2017).

The study illustrates how ambulance staff often had no opportunity to deal with their own distress in relation to suicide-related work and how this can impact on their own wellbeing as well as on how they respond to bereaved families. Some ambulance staff described feeling powerless to help the bereaved and disengaging from them at the scene of a suicide. By contrast, Genest et al. (2018) emphasize that first responder approaches can have an impact on the longer-term resilience of the suicide bereaved; thus increasing the knowledge, skills and confidence of ambulance staff could enable them to provide effective support to the bereaved at the scene of death and help also to increase the bereaved’s longer-term resilience. Training in how to respond to people who are bereaved by suicide more effectively that is informed by ‘self-care’ approaches (Grad, 2012) has demonstrated positive results and could be offered to ambulance staff to empower them (McDonnell et al., 2020). This could not only address gaps in ambulance staff’s expertise in relation to responding to suicide, but and help to meet the previously identified needs of bereaved families (Ross et al., 2019; Wainwright et al., 2019). Furthermore, staff support and training are known to be protective factors by increasing resilience (Castelli Dransart et al., 2017) and could help to reduce suicide risk among ambulance workers.

### Limitations and Strengths

The study is limited by the relatively small, ethnically uniform convenience sample from one ambulance service in England. However, qualitative research seeks not to produce research that is statistically generalizable but to offer insights that are transferable to similar settings. Thus, the themes revealed in the study may be relevant to ambulance services in other parts of the United Kingdom and indeed internationally where similar circumstances are seen to exist. Despite the limited sample, the qualitative approach enabled the gathering of rich and detailed accounts which are fully presented stand as a powerful and credible first exploration of ambulance staff’s perspectives in relation to attending suicides and responding to the bereaved at the scene of death.

### Future Research and Service Development

Given the accounts of high-pressure working described by participants in this study, through large-scale studies, further research should aim to accurately assess the magnitude of the strain on ambulance workers in relation to responding to suicide/bereaved families. Future research could explore the training needs of ambulance staff and other first responders (e.g., police and fire service workers) across countries in relation to responding to deaths by suicide and to bereaved families. Research is urgently needed to identify how employers could support the wellbeing of first responder staff in better ways. Organizational research could explore how first responder services might work together more effectively in responding to suicide in ways that that better support the wellbeing of not only the bereaved but of staff and trainees too.

## Conclusion

The study contributes the in-depth perspectives of ambulance staff who deal with potentially traumatic deaths by suicide. Staff were often anxious and uncertain how to respond to individuals bereaved by suicide at the scene. They also had unmet support needs themselves, were hesitant to access help and coped informally. Training and postvention support for ambulance staff could enable better coping, more effective support for bereaved individuals and reduce the risk of further deaths by suicide within and outside the profession.

## Data Availability Statement

The datasets generated for this study are available on request to the corresponding author.

## Ethics Statement

The studies involving human participants were reviewed and ethical approval was obtained from National Research Ethics Service Committee North West – Greater Manchester West. Research ethics committee reference: 11/NW/21047. The patients/participants provided their written informed consent to participate in this study.

## Author Contributions

PN led on the analysis and interpretation of the study data, drafted the manuscript, revised it critically for intellectual content, provided approval for publication of the content, and agreed to be accountable for all aspects of the work. SM designed the study, recruited participants, collected all study data, jointly analyzed/interpreted the data, revised the manuscript critically for intellectual content, provided approval for publication of the content, and agreed to be accountable for all aspects of the work. LC, NK, CC-G, and JS contributed to the conception and design of the work, revised manuscript critically for intellectual content, provided approval for publication of the content, and agreed to be accountable for all aspects of the work. SS contributed to the acquisition of data, revised the manuscript critically for intellectual content, provided approval for publication of the content, and agreed to be accountable for all aspects of the work. BM revised the manuscript critically for intellectual content, provided approval for publication of the content, and agreed to be accountable for all aspects of the work.

## Disclaimer

The views expressed are those of the author(s) and not necessarily those of the NIHR or the Department of Health and Social Care.

## Conflict of Interest

The authors declare that the research was conducted in the absence of any commercial or financial relationships that could be construed as a potential conflict of interest.
